# Supplementary material for: Factors affecting decision-making in children with complex care needs: a consensus approach to develop best practice in a UK children’s hospital
Source: BMJ Paediatr Open. 2022 Sep 29;6(1):e001589. doi: 10.1136/bmjpo-2022-001589 (PMC9528619; doi:10.1136/bmjpo-2022-001589)
Supplement: Author's manuscript [file bmjpo-2022-001589.draft_revisions.pdf]

BMJ Paediatrics Open

BMJ Paediatrics Open is committed to open peer review. As part of this commitment we make the peer review history of every article we publish publicly available.

When an article is published we post the peer reviewers' comments and the authors' responses online. We also post the versions of the paper that were used during peer review. These are the versions that the peer review comments apply to.

The versions of the paper that follow are the versions that were submitted during the peer review process. They are not the versions of record or the final published versions. They should not be cited or distributed as the published version of this manuscript.

BMJ Paediatrics Open is an open access journal and the full, final, typeset and author-corrected version of record of the manuscript is available on our site with no access controls, subscription charges or pay-per-view fees (http://bmjpaedsopen.bmj.com).

If you have any questions on BMJ Paediatrics Open's open peer review process please email info.bmjpo@bmj.com

BMJ Paediatrics Open

Factors affecting decision making in children with complex care needs: a consensus approach to develop best practice in a UK children's hospital

Journal:	*BMJ Paediatrics Open*
Manuscript ID	bmjpo-2022-001589
Article Type:	Original research
Date Submitted by the Author:	24-Jun-2022
Complete List of Authors:	Birchley, Giles; University of Bristol Medical School, Population Health Sciences<br>Thomas-Unsworth, Sadie; Bristol Royal Hospital for Children, Clinical Psychology<br>Mellor, Charlotte; Bristol Royal Hospital for Children, Paediatric Palliative Care & Bereavement Support<br>Baquedano, Mai; University of Bristol Medical School, Translational Health Sciences<br>Ingle, Susanne; University of Bristol Medical School, Population Health Sciences<br>Fraser, James; Bristol Royal Hospital for Children Paediatric Intensive Care Unit; Paediatric Critical Care Society
Keywords:	Ethics, Qualitative research

SCHOLARONE™
Manuscripts

I, the Submitting Author has the right to grant and does grant on behalf of all authors of the Work (as defined in the below author licence), an exclusive licence and/or a non-exclusive licence for contributions from authors who are: i) UK Crown employees; ii) where BMJ has agreed a CC-BY licence shall apply, and/or iii) in accordance with the terms applicable for US Federal Government officers or employees acting as part of their official duties; on a worldwide, perpetual, irrevocable, royalty-free basis to BMJ Publishing Group Ltd ("BMJ") its licensees and where the relevant Journal is co-owned by BMJ to the co-owners of the Journal, to publish the Work in this journal and any other BMJ products and to exploit all rights, as set out in our licence.

The Submitting Author accepts and understands that any supply made under these terms is made by BMJ to the Submitting Author unless you are acting as an employee on behalf of your employer or a postgraduate student of an affiliated institution which is paying any applicable article publishing charge ("APC") for Open Access articles. Where the Submitting Author wishes to make the Work available on an Open Access basis (and intends to pay the relevant APC), the terms of reuse of such Open Access shall be governed by a Creative Commons licence – details of these licences and which Creative Commons licence will apply to this Work are set out in our licence referred to above.

Other than as permitted in any relevant BMJ Author's Self Archiving Policies, I confirm this Work has not been accepted for publication elsewhere, is not being considered for publication elsewhere and does not duplicate material already published. I confirm all authors consent to publication of this Work and authorise the granting of this licence.

Confidential: For Review Only

Title: Factors affecting decision making in children with complex care needs: a consensus approach to develop best practice in a UK children's hospital

Article type: Original Research

Word count (excluding references and tables): 2325 words

Keywords: Complex Care, Shared Decision Making, Ethics, Policy, Delphi

Author names, addresses and affiliations:

Corresponding author: Dr Giles Birchley

Affiliation: Centre for Ethics in Medicine, Bristol Medical School (PHS), University of Bristol, Bristol, UK.

Email: giles.birchley@bristol.ac.uk

Telephone: +44 117 331 4517

Orcid ID: 0000-0002-2973-2163

Funding: Giles Birchley's research was funded in whole, or in part, by the Wellcome Trust Grant no. 209841/Z/17/Z. The funders had no role in the preparation of this manuscript or the decision to submit for publication. For the purpose of Open Access, the author has applied a CC BY public copyright licence to any Author Accepted Manuscript version arising from this submission.

Dr Sadie Thomas-Unsworth

Affliliation: Bristol Royal Hospital for Children, Bristol, UK

Dr Charlotte Mellor

Affiliation: Bristol Royal Hospital for Children, Bristol, UK

Confidential: For Review Only

Dr Mai Baquedano

Affiliation: Bristol Medical School (THS), University of Bristol, Bristol UK

Dr Suzanne Ingle

Affiliation: Bristol Medical School (PHS), University of Bristol, Bristol UK

Dr James Fraser

Affiliation: 1. Bristol Royal Hospital for Children, Paediatric Intensive Care Unit, Bristol, UK;

2. Paediatric Critical Care Society, London, UK

Conflicts of interests:

The authors have no conflicts of interest to declare.

Confidential: For Review Only

Abstract

Background: Children with complex care needs are a growing proportion of the sick children seen in all healthcare settings in the UK. Complex care needs place demands on parents and professionals that often require many different healthcare teams to work together. Care can be both materially and logistically difficult to manage, causing friction with parents. These difficulties may be reduced if common best practice standards and approaches can be developed in this area.

Objective: To develop a consensus approach to the management of complexity among healthcare professionals, we used a modified delphi process. The process consisted of a meeting of clinical leaders to develop candidate statements followed by two survey rounds open to all professionals in a UK children's hospital to measure and establish consensus recommendations.

Results: 99 Professionals completed both rounds of the survey. 69 statements reached the threshold for consensus. These pertained to five thematic areas; standardised approaches to communicating with families; processes for interprofessional communication; processes for shared decision-making in the child's best interests; role of the multi-disciplinary team; managing professional-parental disagreement and conflict, and; the role of clinical psychologists. Overall the level of consensus was high, ranging from agreement to strong agreement.

Conclusions: These statements provide a consensus basis that can inform standardised approaches to the management of complexity. Such approaches may decrease friction between parents, children and healthcare professionals.

Confidential: For Review Only

Factors affecting decision-making in children with complex care needs: a consensus approach to develop best practice in a UK children's hospital

Confidential: For Review Only

What is already known on this topic: The number of children with complex care needs in UK hospitals is increasing. Difficulties in the management of these children can result in incoordinate decision-making, increased friction with families and a loss of focus on the child's best interests.

What this study adds: A series of statements agreed by a mixed group of professionals at a tertiary children's hospital demonstrates strong clinical consensus about best practice.

How this study might affect research, practice or policy: The statements can be used by healthcare teams to guide better engagement with families, as well as informing standardised policies to reduce friction and improve decision-making.

Introduction

Children's complex care needs have been defined as "multidimensional health and social care needs in the presence of a recognized medical condition or where there is no unifying diagnosis. They are individual and contextualized, are continuing and dynamic, and are present across a range of settings, impacted by healthcare structure."[1] Many children with complex care needs also have life-limiting conditions i.e. a condition for which there is no reasonable hope for cure and from which they will die. The prevalence of children with life-limiting conditions in the UK is increasing.[2]

Decision making in such cases can be medically, socially and ethically complex. Decisions may be misunderstood and/or contested. Disputes, either between different professional teams or between clinicians and the child's family, are common across clinical settings.[3,4] Although the broad legal framework for making decisions has remained relatively consistent for three decades, society has radically changed. Successive NHS reforms have sought to reshape health provision "in the business of customer service".[5] While the welfare of the child remains the "paramount consideration" in law,[6] decisions for and on behalf of children are guided by principles of shared decision making, where the parent and clinician are on a nominally equal footing.[7,8] This welcome recognition of partnership notwithstanding, movements within society assert absolute parental rights to decide what is best for their child's health.[9]

Against this background, advances in medicine mean that increasing numbers of children with complex care needs are surviving for longer and occupying a greater proportion of hospital bed-days.[10] Invariably such patients fall under numerous medical teams and social agencies, each trying to serve the needs and wishes of the child and their family. Commonly, a lack of dedicated leadership results in incoordinate decision making, the family confused, and the best interests of the child overlooked. Such factors might exacerbate professional-parental disharmony and contribute to complaints and litigation, while compromising the wellbeing of all concerned parties.[11]

While much has been written on how best to manage conflict in paediatric practice,[12,13] there is less consensus on the best approach to decision-making in children with complex needs. Best practice

should help clinicians to compassionately deliver high quality, medically appropriate clinical care, while successfully collaborating with children, families and colleagues.

Method

We used a modified Delphi process to determine consensus across key professional groups.[14] An initial face-to-face meeting of selected clinical leaders (doctors, nurses, managers) was arranged to identify the key themes underpinning decision making. Participants received short lectures on essential law and ethics, the evidence base for partnership working, and the factors contributing to conflict. There followed 3 facilitated discussions. In the first, five groups of participants, each containing 6-8 professionals from mixed disciplinary and clinical backgrounds, shared experiences amongst themselves of managing complexity. In the second, groups fed back to one another the problems faced and approaches used in their own practice. Finally, participants developed consensus opinions around key themes, which were then developed after the meeting by CM, STU, GB, and JF into normative 'statements' around standards and approaches to managing complexity. The resultant themes and statements were finally shared back with the meeting participants who were invited to check them for accuracy. In two cases additional statements were suggested by participants. These were checked against existing themes and incorporated into the supporting statements.

Two sequential Delphi rounds were used to rank supporting statements within each theme. Participants scored their agreement with each statement using a 5 -point Likert scale: 1 = strongly disagree, 2 = disagree, 3 = neutral, 4 = agree, 5 = strongly agree. Participants also had the option to select 6 = don't know. Invitations to take part in the survey were sent by email to multi-professional staff members across the Children's hospital. This survey involved senior managers, all medical consultants, all ward nursing managers, all clinical nurse specialists (CNS), all psychologists, senior allied healthcare professionals (AHP), family liaison team members and the chaplaincy. The email invitation directed each staff member to a RedCap portal where they registered their professional group, level of experience, and were assigned a unique identifying survey number. After 16 weeks the survey was closed. Consensus for support of a particular statement was deemed to occur when, after excluding scores indicating 'don't know', 70% of respondents scored that statement >= 3. These statements were carried forward to the second round.

All participants who participated in the first round of the survey were then emailed an invitation to participate in a second Delphi round. In the second round, participants were asked to again score their agreement with each ranked statement from round 1. In round 2, they were additionally provided with the median score for each statement from all participants, alongside their own score, from round 1. The final scores for each statement, from all participants in round 2, were analysed to determine their mean, median and interquartile ranges.

Patients and the public were not involved in the design or reporting of this research.

Statistical analysis

Items that were scored 6 (=don't know) were treated as missing data and not included in the statistical analysis. Inspection of bar charts determined that scores were not normally distributed. Therefore, medians and Interquartile ranges (25th percentile to 75th percentile) were used to measure central tendency.

Chi-squared tests were used to determine the strength of evidence for any differences ($p<0.1$) between the median scores of participants who had dropped out between Delphi rounds one and

Confidential: For Review Only

two compared with those who completed both round. Non-parametric K-sample tests were used to compare median responses across different professional backgrounds and years of experience. The large number of statements being compared (69) could lead to small p-values purely by chance. To avoid this problem of multiple testing we used the Bonferroni correction to adjust the p-value of interest to be 0.0014 (0.1/69). Therefore, no association was deemed noteworthy unless it had a p<=0.0014.

Ethics

The study was formally registered with the University Hospitals Quality Improvement 'hub' as a service development project, so formal ethics approval was not required.

Results

The initial consensus forming face to face meeting proposed 77 normative statements grouped into 7 key themes (themes summarised in table 1 and full listing of normative statements in table 4).

Consensus group themes
**Standardised approaches to communicating with families**
**Processes for interprofessional communication**
**Processes for shared decision-making in the child's best interests**
**Role of the multi-disciplinary team**
**Managing professional-parental disagreement and conflict**
**The role of clinical psychologists**
**Processes to support staff**

Table 1: Themes

In the first Delphi round, surveys were sent to 390 professionals and 163 (42%) replied. In the second Delphi round the 163 professionals who had participated in the first round were surveyed again, and 99 (60%) returned surveys with all delphi items scored. Across the 69 delphi items answered by 99 particpants there were only 66 responses (0.9%) of 6 =don't know. There was no statistical difference between the responses of those participants who only responded to round 1 of the Delphi survey and those who responded to both rounds 1 and 2.

The professional background of the 99 participants is shown in Table 2.

Background of Participants	
**Professional background**	Number of participants
**Consultant**	46
**Senior nurse/ ward manager**	16
**Clinical nurse specialist**	25
**Psychology**	5
**AHP**	3
**Family support service**	2
**Chaplain**	1
**Unknown (did not answer)**	1
Total	*99*

Table 2: Participant professional background

Statistical analysis showed reasonable evidence that professional background affected the median value being assigned for 2 normative statements. Statement 5 (table 4) was more strongly supported

Confidential: For Review Only

by those from a consultant, CNS, or ‘other’ background, while statement 56 (table 4) was more strongly supported by those from a psychology backgrounds.

The years of experience of the participants is shown in Table 3.

**Participant years of experience**	
**Years of experience**	Number of participants
**< 2**	11
**2-5**	21
**5-10**	23
**> 10**	43
**Unknown (did not answer)**	1
Total	*99*

Table 3: Participant years of experience

Statistical analysis showed little evidence that years of experience affected the median values for any of the normative statements.

All normative statements met the a priori consensus *(>70% of respondents scoring each statement greater than or equal to 3*) for proceeding to the second Delphi round. In the second round, while the mean score for each statement ranged from 3.0 (neutral) to 4.9 (strong agreement) (see detail in Table 4), there was little evidence of differences across median scores in the manner participants ranked their responses, either across themes or within themes.

Confidential: For Review Only

Theme	No.	Statement	Mean score	Standard deviation	Median	IQR 25	IQR 75
When communicating with families a standardised approach should be applied to…	1	*Agreeing the message communicated to families to avoid giving conflicting ambiguous messages*	4.4	0.8	4	4	5
	2	*Assessing the families concerns so as to ensure they are directed to the right person in any discussion*	4.9	0.4	5	5	5
	3	*Noting discussions*	4.6	0.7	5	4	5
	4	*Ensuring the preceding antenatal consultations are shared among the treating team in a coordinated manner*	4.9	0.4	5	5	5
	5	*Acknowledging the contribution of all professionals from Consultant to bedside nurse to junior doctor*	4.6	1.1	5	4	5
	6	*Information sharing among professionals*	4.8	0.5	5	5	5
	7	*Using 'family held records' to allow families to document meetings , their content, and reflections of understanding*	4.1	0.7	4	4	5
When communicating between professionals…	8	*Processes should be put in place by the hospital Trust to strengthen communication between community care providers, hospices, and regional hospitals*	4.6	0.6	5	4	5
	9	*Professionals in the 'team around the child' should be acknowledged and identified early in the child's care pathway*	4.7	0.5	5	4	5
	10	*A case worker/ liaison nurse should be assigned to each complex case to improve coordination across different teams*	4.7	0.6	5	4	5
	11	*A single clinical lead for the child should be defined across all the involved speciality teams*	4.7	0.5	5	4	5
In the process of shared decision	12	*We need to be honest with families about medical uncertainty*	4.9	0.3	5	5	5

Confidential: For

Theme	No.	Statement	Mean score	Standard deviation	Median	IQR 25	IQR 75
making in the child's best interests…	*13*	*There should be a holistic assessment of the child's and families wishes, values and goals to inform more complex decisions later*	4.4	0.7	5	4	5
	14	*We need to, where possible and appropriate, need to listen to what children have to say*	3.7	1.0	4	3	4
	15	*Be aware that children may have motivations separate to what they may say (eg wanting to please their parents)*	4.6	1.0	5	4	5
	16	*Children have rights and these may require independent advocacy even if this means health care professionals disagreeing with their parents*	4.8	0.4	5	5	5
	17	*A holistic assessment of the child's and families wishes, values and goals should include an assessment of their spiritual and religious beliefs*	4.8	0.4	5	5	5
	18	*We should engage families in 'parallel planning' early and routinely in a child's disease course*	4.8	0.5	5	5	5
	19	*Professionals need training to understand the legal framework in which they operate*	4.8	0.4	5	5	5
	20	*Professionals needs to be trained in communication techniques*	4.7	0.5	5	4	5
	21	*A holistic assessment of the child's and families wishes, values and goals should be done in partnership with members of the child's MDT*	4.5	0.7	5	4	5
	22	*We should try to provide options rather than making closed recommendations*	4.5	0.8	5	4	5
	23	*Professionals find it distressing when they are unable to fix medical problems and need supporting when this happens*	4.3	0.8	4	4	5
	24	*We need to transparently share our own values and*	4.5	0.7	5	4	5

Confidential: For Review Only

Theme	No.	Statement	Mean score	Standard deviation	Median	IQR 25	IQR 75
		goals with families and children					
The MDT should…	*25*	*Be well supported through administrative assistance with preparation and note taking*	4.4	0.6	4	4	5
	26	*Have outcomes recorded in a consistent and transparent fashion*	4.3	0.8	4	4	5
	27	*Be recognised in job plans, given that repeated attendance at MDT meetings by recognised key professionals is onerous and takes time*	4.6	0.6	5	4	5
	28	*Be held in an appropriate physical environment to enable clarity of discussion*	4.8	0.4	5	5	5
	29	*Be attended by the wider team including hospital community . and hospice representatives*	3.6	1.0	4	3	4
	30	*Ensure that appropriate weight given to all expressed views*	3.0	0.8	3	3	3
	31	*Be chaired by professionals who are trained in chairing such meetings*	4.7	0.5	5	4	5
	32	*Only take place when attempts have been made to understand the child's values and goals*	4.4	0.7	4	4	5
	33	*Be chaired by a professional outside the child's primary clinical team*	4.1	0.7	4	4	5
When managing professional – parental disagreement or conflict…	*34*	*Families should be given realistic honestly held opinions*	4.3	0.7	4	4	5
	35	*We need to better recognise and support mental health issues in families*	4.3	0.7	4	4	5
	36	*We need to prevent the 'threat response' whereby professionals adopt behaviours to avoid contact with families/ each other*	4.8	0.4	5	5	5
	37	*The organisation should recognise that (in a rights and consumer based society) any framework that is put in*	4.7	0.8	5	4	5

Only

Theme	No.	Statement	Mean score	Standard deviation	Median	IQR 25	IQR 75
		place to improve decision making may not negate conflict and/ or complaint					
	38	*Professional-parental disagreement takes resources and time from other patients and so should be an issue of the highest priority for the organisation*	4.1	0.8	4	4	5
	39	*The organisation has a responsibility to convey the challenges of decision making, in the context of patient complexity, to the wider society (local community, NHS leaders, national bodies)*	3.5	1.1	4	3	4
	40	*Families and professionals should receive advice and support on the benefits and risks of social media use*	4.0	1.1	4	4	4
	41	*Standardised information should be provided to families explaining how the decision making process works*	3.6	0.8	4	3	4
	42	*In rare circumstances parental-behavioural contracts are an important tool in addressing disruptive parental behaviour*	3.7	0.8	4	3	4
	43	*A standardised pathway for decision making should set out processes for where disagreement arises*	4.0	1.1	4	4	4
	44	*The CEAG is an important resource and should be included in any standardised pathway*	3.8	1.3	4	3	4
	45	*There is a need nationally to standardise processes by which 2nd opinions are sought (when?, who? how?, with parental engagement?)*	4.1	1.1	4	4	5
	46	*External second opinions should be sought from a national peer reviewed speciality MDT where these are available*	4.3	0.6	4	4	5
	47	*The ward round hand over is an important opportunity to identify evolving issues*	4.7	0.5	5	4	5

Confidential: For Review Only

Theme	No.	Statement	Mean score	Standard deviation	Median	IQR 25	IQR 75
	48	*National peer reviewed MDTs should be convened in specialities where they do not exist*	4.3	1.0	4	4	5
	49	*Gaining an external second opinion is important and should be included in any standardised pathway*	4.4	0.6	4	4	5
	50	*Gaining a local second opinion is important and should be included in any standardised pathway*	4.3	0.8	4	4	5
	51	*A 'traffic light system' whereby a family's behaviour is graded allows earlier identification of conflict and should be included in a standardised pathway to reduce conflict*	4.4	1.0	4	4	5
Clinical psychologists should…	*52*	*Be integrated within all clinical teams*	4.3	1.0	4	4	5
	53	*Support the decision-making process*	4.5	1.0	5	4	5
	54	*Support training in communication techniques*	4.2	1.0	4	4	5
	55	*Routinely explore family goals and values and explicitly share such information with the MDT*	4.4	0.9	4	4	5
	56	*Have their role better explained to families*	4.4	0.9	4	4	5
	57	*Support training n chairing complex MDT meetings*	4.2	1.0	4	4	5
	58	*Be involved from the outset in all complex decision-making discussions*	4.4	1.0	5	4	5
In supporting staff…	*59*	*Teams need to adopt process where they can come together to discuss challenging cases*	4.7	0.5	5	4	5
	60	*Teams need to adopt process where individual colleagues are supported*	4.8	0.4	5	5	5
	61	*The organisation should recognise that parental-professional disagreement (conflict) results in poor morale and staff attrition*	4.8	0.5	5	5	5
	62	*The organisation should recognise the physical, mental, and reputational harm done to professionals in extreme cases of professional-parental disharmony*	4.8	0.4	5	5	5

Confidential: For Review Only

Theme	No.	Statement	Mean score	Standard deviation	Median	IQR 25	IQR 75
	63	*The organisation should recognise that nursing colleagues are particularly vulnerable due to the requirement for them to be at the bedside 24 hours a day*	4.9	0.4	5	5	5
	64	*The organisation should recognise that HCPs intrinsic desire to 'do the right thing' through leading in complex cases often overrides regard for their personal wellbeing, and increases their vulnerability to experiencing moral distress, compassion fatigue and burnout*	4.2	1.0	4	4	5
	65	*Professionals should take decisions as a team seeking quoracy (consensus within the core team) wherever possible*	4.6	0.6	5	4	5
	66	*Staff have a responsibility themselves to access available support options*	4.2	0.7	4	4	5
	67	*Cross speciality case forums (such as Schwartz rounds) are helpful for different teams to come together to discuss challenging scenarios. While not part of a decision making pathway they should be available for staff aftercare.*	3.8	0.8	4	3	4
	68	*The organisation should provide better opportunities for 1:1 and group peer support*	4.1	0.8	4	4	5
	69	*The organisation should do more to signpost colleagues to available resources*	4.8	0.4	5	5	5

Table 4: Results after final round of Delphi

Confidential: For Review Only

Discussion

The most notable finding in our study was the uniform degree of consensus across a group of hospital professionals on how best to approach decision making in children with complex care needs. The 5 major themes (standardising the approach to communication with families, communication between professionals, shared decision making, the role of the multidisciplinary team, managing conflict, the role of clinical psychology, and staff wellbeing) resonated with colleagues and provided a useful framework for exploring specific principles. While the level of consensus created by the discussions in the workshop and discussions and subsequently measured in all themes was striking, there is perhaps some merit for observing those that carried weight and discussing their implications.

The survey highlights the essential importance of good communication both with the family and between professionals. Concepts such as the 'team around the child' , a liaison or 'key worker', and a single defined clinical lead, are not new concepts but do require some committed resource and intent. All too often parental-professional disharmony arises due to misunderstandings or perceived poor co-ordination of care. While the survey talks of a 'standardised' approach to communication, this is not intended to imply a 'one size fits all' solution. Each family is unique and good communication is successful because the needs of each participant are individually recognised.[15] There is a delicate balance between structure and intimate spontaneity that must be achieved in practice.

There was strong agreement that clinicians and parents should work in partnership for the best interests of the child. This is particularly pertinent in the wake of the Charlie Gard case[16] where some voices within academic ethical and legal circles have argued that a shift in authority toward parents in shared decision-making is needed.[17] Participants supported the principle that children have rights that might require independent advocacy. However, while there was strong consensus that transparency was important, professionals were more ambivalent about the importance of this reflecting their own values and goals (statements 24). Finally, there was consensus that good care for children might imply a willingness to consider the child in isolation as well as within a family unit, and that one should not automatically assume that children will share their parents' values.

There was strong agreement about the importance and structure of the MDT although there was a predominant view that such a meeting need not be chaired by a professional outside the child's primary clinical team (statement 33). Interestingly there was some ambivalence expressed as to whether understanding the child's values and goals should automatically precede such meetings (statement 33) although this might reflect a pragmatic view around the logistics of holding such meetings. MDT meetings with or without families form the vital platform upon which clinical decisions are taken. They involve multiple professionals, require much planning, and yet, due to their 'ad hoc nature', do not appear in job plans or have sufficient resource.

When things go wrong, complaint and conflict too often arise. These are challenging for both families and members of the clinical team. There was a tacit acknowledgment that, despite best intentions and optimal circumstances, conflict will occasionally still arise. Organisations should give conflict resolution the highest priority and consider standardised approaches to clinical ethics consultation and external second opinions. Referral for external second opinions has been a consistent recommendation for reducing conflict in many previous publications.[12,18,19] However, in our study, while there was a strong consensus for such processes to be standardised at a national

Confidential: For Review Only

level (statement 45), there was slightly more ambivalence around these being introduced as a component of an automatic conflict resolution pathway at a local level (statement 49 & 50). Participants in our study were also supportive of involving national peer review MDTs where these were available (statement 46) or establishing such bodies where they were not (statement 48).

There was consensus for Clinical Psychology support to be integrated within clinical teams, and for them to take an active role in exploring family goals and values and supporting complex decision-making discussions. Finally, there was strong agreement around the importance of supporting staff, and that, in situations of professional-parental disharmony, poor morale and staff attrition result. The deleterious impact on clinicians of difficult relationships and challenging dilemmas in practice has been previously noted.[20,21,22] A primary focus on individual resilience may miss the importance of investment in institution wide structures.[23] Health care professionals' intrinsic desire to 'do the right thing' through leading in complex cases may over-ride their regard for their personal wellbeing, and increase their vulnerability to experiencing moral distress, compassion fatigue and burnout.

Limitations

Importantly, we did not consider the views of either parents or children in this delphi survey. Further consensus work will need to ensure that the statements included here are a solid basis of a model for partnership between hospital staff, parents and children. Given the high degree of consensus shown, it is worth considering if this consensus was genuine. Our statistical analysis showed that dropping out of the delphi was not due to being a statistical outlier, suggesting the process did not discourage dissenters from taking part. Nevertheless, 74.5% of those approached did not engage with the process at all. Although the level of engagement seems broadly comparable with similar studies that published this data[24,25] (and accounting for the viewpoints of those who do not engage with research is a problem common to all types of research) we cannot discount the idea that a proportion of these staff did not value the aims of the study.

Conclusion

The number of children with complex care needs in our hospitals is increasing. Our study of a large mixed group of healthcare professionals in a tertiary children's hospital demonstrates strong consensus across 5 key themes towards developing best practice in decision making in children with complex care needs: standardising the approach to communication with families, communication between professionals, shared decision making, the role of the multidisciplinary team, managing conflict, the role of clinical psychology, and staff wellbeing. Within each of these themes there are notable elements of practice that health care teams might engage with to better support the patients and families in their care. In some situations, a comprehensive adoption of a standardised approach, might mitigate against professional-parental disharmony.

Confidential: For Review Only

References:

[1] Brenner M, Kidston C, Hilliard C, *et al.* Children's complex care needs: a systematic concept analysis of multidisciplinary language. *Eur J Pediatr*. 2018;177(11):1641-1652.

[2] Fraser LK, Gibson-Smith D, Jarvis, S *et al*. Estimating the current and future prevalence of life-limiting conditions in children in England. *Palliat Med*. 2021;35(9):1641-1651.

[3] Forbat L, Sayer C, McNamee P, *et al.* Conflict in a paediatric hospital: a prospective mixed-method study. *Arch Dis Child* 2016; 101(1):23-7
[4] Azoulay E, Timsit JF, Sprung CL. Prevalence and factors of intensive care unit conflicts: the conflicus study. *Am J Respir Crit Care Med* 2009;180:853-60
[5] Department of Health (2003) Building on the Best: Choice, Responsiveness and Equity in the NHS, Cmnd. 6079 (London, The Stationery Office): 63
[6] Children Act 1989 s.1(1)
[7] Streuli JC, Anderson J, Alef-Defoe S, *et al.* Combining the best interest standard with shared decision-making in paediatrics—introducing the shared optimum approach based on a qualitative study. *Eur J Pediatr* 2021;180**:** 759–766
[8] Birchley G, Deciding together? Best interests and shared decision-making in paediatric intensive care. *Health Care Anal* 2014; 22:203–222
[9] The Gard Foundation, What is Charlie's Law? Available at: https://thecharliegardfoundation.org/ Accessed 25th April 2022
[10] Kanthimathinathan HK, Plunkett A, Scholefield BR *et al.* Trends in long-stay admissions to a UK paediatric intensive care unit *Arch Dis Child* 2020;105:558-562
[11] Gerada C (editor), *Beneath the White Coat: Doctors, Their Minds and Mental Health* (Routledge 2020)
[12] Linney M, Hain RDW, Wilkinson D, *et al* Achieving consensus advice for paediatricians and other health professionals: on prevention, recognition and management of conflict in paediatric practice *Arch Dis Child* 2019; 104:413-416.
[13] Forbat L, Teuten B, Barclay S, Conflict escalation in paediatric services: findings from a qualitative study *Arch Dis Child* 2015;100:769-773.
[14] Murphy MK , Black NA , Lamping DL, *et al.* Consensus development methods, and their use in clinical Guideline development. *Health Technol Assess* 1998; 2:i–iv.1-88.
[15] Ward P. Trust and communication in a doctor-patient relationship: a literature review. *Arch Med.* 2018;3(3):36.
[16] *Yates and Gard v Great Ormond Street Hospital* [2017] EWCA Civ 410
[17] Auckland, C and Goold, I (2020) Re-evaluating 'best interests' in the wake of Raqeeb v Barts NHS Foundation Trust & Anors *Mod Law Rev* 83(6) 1328-1342
[18] Austin L, Huxtable R. Resolving Disagreements about the Care of Critically Ill Children: Evaluating Existing Processes and Setting the Research Agenda In: Goold I, Herring J, Auckland C (eds) Parental Rights, Best Interests and Significant Harms: Medical Decision-Making on Behalf of Children Post-Great Ormond Street Hospital v Yates (Oxford: Hart Publishing, 2019)
[19] Nuffield Council on Bioethics, Disagreements in the care of critically ill children (Bioethics Briefing Note) (London: Nuffield Council on Bioethics, 2019)
[20] Gerada C (editor), Beneath the White Coat: Doctors, Their Minds and Mental Health (Routledge 2020); Barclay S. Recognizing and managing conflict between patients, parents and health professionals. *Paediatr Child Health* 2016; 26(7):314-315
[21] Carmela Mento, Maria Catena Silvestri, Antonio Bruno, *et al.* Workplace violence against healthcare professionals: A systematic review, *Aggress Violent Behav* 2020; 51:101381
[22] Knickle K, McNaughton N, Downar J. Beyond winning: mediation, conflict resolution, and non-rational sources of conflict in the ICU. *Crit Care* 2012; 16:308
[23] Johal HK, Prout R, Birchley G *et al.* We should move away from a focus on individual resilience towards building resilient systems *BMJ* 2021; 375:2737
[24] Murray MJ, Ruffle A, Lowis S, *et al* Delphi method to identify expert opinion to support children's cancer referral guidelines *Arch Dis Child* 2020; 105:241-246
[25] Ekberg S, Herbert A, Johns K, *et al.* Finding a way with words: Delphi study to develop a discussion prompt list for paediatric palliative care. *Palliat Med* 2020; 34(3):291-299

Confidential: For Review Only

BMJ Paediatrics Open

Factors affecting decision making in children with complex care needs: a consensus approach to develop best practice in a UK children's hospital

Journal:	*BMJ Paediatrics Open*
Manuscript ID	bmjpo-2022-001589.R1
Article Type:	Original research
Date Submitted by the Author:	05-Sep-2022
Complete List of Authors:	Birchley, Giles; University of Bristol Medical School, Population Health Sciences<br>Thomas-Unsworth, Sadie; Bristol Royal Hospital for Children, Clinical Psychology<br>Mellor, Charlotte; Bristol Royal Hospital for Children, Paediatric Palliative Care & Bereavement Support<br>Baquedano, Mai; University of Bristol Medical School, Translational Health Sciences<br>Ingle, Susanne; University of Bristol Medical School, Population Health Sciences<br>Fraser, James; Bristol Royal Hospital for Children Paediatric Intensive Care Unit; Paediatric Critical Care Society
Keywords:	Ethics, Qualitative research

SCHOLARONE™
Manuscripts

BMJ

I, the Submitting Author has the right to grant and does grant on behalf of all authors of the Work (as defined in the below author licence), an exclusive licence and/or a non-exclusive licence for contributions from authors who are: i) UK Crown employees; ii) where BMJ has agreed a CC-BY licence shall apply, and/or iii) in accordance with the terms applicable for US Federal Government officers or employees acting as part of their official duties; on a worldwide, perpetual, irrevocable, royalty-free basis to BMJ Publishing Group Ltd ("BMJ") its licensees and where the relevant Journal is co-owned by BMJ to the co-owners of the Journal, to publish the Work in this journal and any other BMJ products and to exploit all rights, as set out in our licence.

The Submitting Author accepts and understands that any supply made under these terms is made by BMJ to the Submitting Author unless you are acting as an employee on behalf of your employer or a postgraduate student of an affiliated institution which is paying any applicable article publishing charge ("APC") for Open Access articles. Where the Submitting Author wishes to make the Work available on an Open Access basis (and intends to pay the relevant APC), the terms of reuse of such Open Access shall be governed by a Creative Commons licence – details of these licences and which Creative Commons licence will apply to this Work are set out in our licence referred to above.

Other than as permitted in any relevant BMJ Author's Self Archiving Policies, I confirm this Work has not been accepted for publication elsewhere, is not being considered for publication elsewhere and does not duplicate material already published. I confirm all authors consent to publication of this Work and authorise the granting of this licence.

Confidential: For Review Only

Title: Factors affecting decision making in children with complex care needs: a consensus approach to develop best practice in a UK children's hospital

Article type: Original Research

Word count (excluding references and tables): 2848 words

Keywords: Complex Care, Shared Decision Making, Ethics, Policy, Delphi

Author names, addresses and affiliations:

Corresponding author: Dr Giles Birchley

Affiliation: Centre for Ethics in Medicine, Bristol Medical School (PHS), University of Bristol, Bristol, UK.

Email: giles.birchley@bristol.ac.uk

Telephone: +44 117 331 4517

Orcid ID: 0000-0002-2973-2163

Funding: Giles Birchley's research was funded in whole, or in part, by the Wellcome Trust Grant no. 209841/Z/17/Z. The funders had no role in the preparation of this manuscript or the decision to submit for publication. For the purpose of Open Access, the author has applied a CC BY public copyright licence to any Author Accepted Manuscript version arising from this submission.

Dr Sadie Thomas-Unsworth

Affliliation: Bristol Royal Hospital for Children, Bristol, UK

Dr Charlotte Mellor

Affiliation: Bristol Royal Hospital for Children, Bristol, UK

Confidential: For Review Only

Dr Mai Baquedano

Affiliation: Bristol Medical School (THS), University of Bristol, Bristol UK

Dr Suzanne Ingle

Affiliation: Bristol Medical School (PHS), University of Bristol, Bristol UK

Dr James Fraser

Affiliation: 1. Bristol Royal Hospital for Children, Paediatric Intensive Care Unit, Bristol, UK;

2. Paediatric Critical Care Society, London, UK

Conflicts of interests:

The authors have no conflicts of interest to declare.

Confidential: For Review Only

Abstract

Background: Children with complex care needs are a growing proportion of the sick children seen in all healthcare settings in the UK. Complex care needs place demands on parents and professionals that often require many different healthcare teams to work together. Care can be both materially and logistically difficult to manage, causing friction with parents. These difficulties may be reduced if common best practice standards and approaches can be developed in this area.

Objective: To develop a consensus approach to the management of complexity among healthcare professionals, we used a modified delphi process. The process consisted of a meeting of clinical leaders to develop candidate statements followed by two survey rounds open to all professionals in a UK children's hospital to measure and establish consensus recommendations.

Results: 99 Professionals completed both rounds of the survey. 69 statements were agreed. These pertained to five thematic areas; standardised approaches to communicating with families; processes for interprofessional communication; processes for shared decision-making in the child's best interests; role of the multi-disciplinary team; managing professional-parental disagreement and conflict, and; the role of clinical psychologists. Overall the level of consensus was high, ranging from agreement to strong agreement.

Conclusions: These statements provide a consensus basis that can inform standardised approaches to the management of complexity. Such approaches may decrease friction between parents, children and healthcare professionals.

Confidential: For Review Only

Factors affecting decision-making in children with complex care needs: a consensus approach to develop best practice in a UK children's hospital

What is already known on this topic: The number of children with complex care needs in UK hospitals is increasing. Difficulties in the management of these children can result in incoordinate decision-making, increased incidence of disagreements with families and a loss of focus on the child's best interests.

What this study adds: A series of statements agreed by a mixed group of professionals at a tertiary children's hospital demonstrates strong clinical consensus about best practice.

How this study might affect research, practice or policy: The statements can be used by healthcare teams to guide better engagement with families, as well as informing standardised policies to reduce incidence of disagreements and improve decision-making.

Confidential: For Review Only

Introduction

Children's complex care needs have been defined as "multidimensional health and social care needs in the presence of a recognized medical condition or where there is no unifying diagnosis. They are individual and contextualized, are continuing and dynamic, and are present across a range of settings, impacted by healthcare structure."[1] Many children with complex care needs also have life-limiting conditions i.e. a condition for which there is no reasonable hope for cure and from which they will die. The prevalence of children with life-limiting conditions in the UK is increasing.[2]

Decision making in such cases can be medically, socially and ethically complex. Decisions may be misunderstood and/or contested. Disputes, either between different professional teams or between clinicians and the child's family, are common across clinical settings.[3,4] Although the broad legal framework for making decisions has remained relatively consistent for three decades, society has radically changed. Successive NHS reforms have sought to reshape health provision "in the business of customer service".[5] While the welfare of the child remains the "paramount consideration" in law,[6] decisions for and on behalf of children are guided by principles of shared decision making, where the parent and clinician are on a nominally equal footing.[7,8] This welcome recognition of partnership notwithstanding, movements within society assert absolute parental rights to decide what is best for their child's health.[9]

Against this background, advances in medicine mean that increasing numbers of children with complex care needs are surviving for longer and occupying a greater proportion of hospital bed-days.[10] Invariably such patients fall under numerous medical teams and social agencies, each trying to serve the needs and wishes of the child and their family. Commonly, a lack of dedicated leadership results in incoordinate decision making, the family confused, and the best interests of the child overlooked. Such factors might exacerbate professional-parental disharmony and contribute to complaints and litigation, while compromising the wellbeing of all concerned parties.[11]

While much has been written on how best to manage conflict in paediatric practice,[12,13] there is less consensus on the best approach to decision-making in children with complex needs. Best practice should help clinicians to compassionately deliver high quality, medically appropriate clinical care, while successfully collaborating with children, families and colleagues.

Method

We used a modified Delphi process to determine consensus across key professional groups.[14] An initial face-to-face meeting of selected clinical leaders (doctors, nurses, managers) was arranged to identify the key themes underpinning decision making. Participants received short lectures on essential law and ethics, the evidence base for partnership working, and the factors contributing to conflict. There followed 3 facilitated discussions. In the first, five groups of participants, each containing 6-8 professionals from mixed disciplinary and clinical backgrounds, shared experiences amongst themselves of managing complexity. In the second, groups fed back to one another the problems faced and approaches used in their own practice. Finally, participants developed consensus opinions around key themes, which were then developed after the meeting by CM, STU, GB, and JF into normative 'statements' around standards and approaches to managing complexity. The resultant themes and statements were finally shared back with the meeting participants who were invited to check them for accuracy. In two cases additional statements were suggested by participants. These were checked against existing themes and incorporated into the supporting statements.

Two sequential Delphi rounds were used to rank supporting statements within each theme. Participants scored their agreement with each statement using a 5 -point Likert scale: 1 = strongly disagree, 2 = disagree, 3 = neutral, 4 = agree, 5 = strongly agree. Participants also had the option to select 6 = don't know. Invitations to take part in the survey were sent by email to multi-professional staff members across the Children's hospital. This survey involved senior managers, all medical consultants, all ward nursing managers, all clinical nurse specialists (CNS), all psychologists, senior allied healthcare professionals (AHP), family liaison team members and the chaplaincy. The email invitation directed each staff member to a RedCap portal where they registered their professional group, level of experience, and were assigned a unique identifying survey number. After 16 weeks the survey was closed. Agreement that a particular statement should be supported and carried forward to the second round was based upon the following: 70% of respondents had scored that statement >= 3 with exclusion of those who had indicated 'don't know'.

All participants who participated in the first round of the survey were then emailed an invitation to participate in a second Delphi round. In the second round, participants were asked to again score their agreement with each ranked statement from round 1. In round 2, they were additionally provided with the median score for each statement from all participants, alongside their own score, from round 1 to explicitly allow participants to revise their scores on the strength of emerging consensus. The final scores for each statement, from all participants in round 2, were analysed to determine their mean, median and interquartile ranges.

Patients and the public were not involved in the design or reporting of this research for reasons that are explained in the limitations section of this article.

Statistical analysis

Items that were scored 6 (=don't know) were treated as missing data and not included in the statistical analysis. We inspected bar charts in order to assess the most appropriate measure of normality. This provided highly compelling evidence that scores were not normally distributed.

Confidential: For Review Only

Therefore, medians and Interquartile ranges (25th percentile to 75th percentile) were used to measure central tendency.

Chi-squared tests were used to determine the strength of evidence for any differences (p<0.1) between the median scores of participants who had dropped out between Delphi rounds one and two compared with those who completed both round. We used a p of 0.1 in order to gauge whether there may have been some weak associations in the data which may have otherwise been dismissed due to the relatively small sample size, rather than as an arbitrary cut point of significance.[15] Non-parametric K-sample tests were used to compare median responses across different professional backgrounds and years of experience. The large number of statements being compared (69) could lead to small p-values purely by chance. To avoid this problem of multiple testing we used the Bonferroni correction to adjust the p-value of interest to be 0.0014 (0.1/69). Therefore, no association was deemed noteworthy unless it had a p<=0.0014.

Ethics

The study was formally registered with the University Hospitals Quality Improvement 'hub' as a service development project, so formal ethics approval was not required.

Results

The initial consensus forming face to face meeting proposed 77 normative statements grouped into 7 key themes (themes summarised in table 1 and full listing of normative statements in table 4).

Consensus group themes
**Standardised approaches to communicating with families**
**Processes for interprofessional communication**
**Processes for shared decision-making in the child's best interests**
**Role of the multi-disciplinary team**
**Managing professional-parental disagreement and conflict**
**The role of clinical psychologists**
**Processes to support staff**

Table 1: Themes

In the first Delphi round, surveys were sent to 390 professionals and 163 (42%) replied. In the second Delphi round the 163 professionals who had participated in the first round were surveyed again, and 99 (60%) returned surveys with all Delphi items scored. Across the 69 Delphi items answered by 99 participants there were only 66 responses (0.9%) of 6 =don't know. There was no statistical difference between the responses of those participants who only responded to round 1 of the Delphi survey and those who responded to both rounds 1 and 2.

The professional background of the 99 participants is shown in Table 2.

Background of Participants	
**Professional background**	Number of participants
**Consultant**	46
**Senior nurse/ ward manager**	16
**Clinical nurse specialist**	25
**Psychology**	5
**AHP**	3
**Family support service**	2

Confidential: For Review Only

**Chaplain**	1
**Unknown (did not answer)**	1
Total	*99*

Table 2: Participant professional background

Statistical analysis showed reasonable evidence that professional background affected the median value being assigned for 2 normative statements. Statement 5 (table 4) was more strongly supported by those from a consultant, CNS, or 'other' background, while statement 56 (table 4) was more strongly supported by those from a psychology backgrounds.

The years of experience of the participants is shown in Table 3.

**Participant years of experience**	
**Years of experience**	Number of participants
**< 2**	11
**2-5**	21
**5-10**	23
**> 10**	43
**Unknown (did not answer)**	1
Total	*99*

Table 3: Participant years of experience

Statistical analysis showed little evidence that years of experience affected the median values for any of the normative statements.

All normative statements met the a priori consensus *(>70% of respondents scoring each statement greater than or equal to 3*) for proceeding to the second Delphi round. In the second round, while the mean score for each statement ranged from 3.0 (neutral) to 4.9 (strong agreement) (see detail in Table 4), there was little evidence of differences across median scores in the manner participants ranked their responses, either across themes or within themes.

Confidential: For Review Only

Theme	No.	Statement	Mean score	Standard deviation	Median	IQR 25	IQR 75
When communicating with families a standardised approach should be applied to…	*1*	*Agreeing the message communicated to families to avoid giving conflicting ambiguous messages*	4.4	0.8	4	4	5
	2	*Assessing the families concerns so as to ensure they are directed to the right person in any discussion*	4.9	0.4	5	5	5
	3	*Noting discussions*	4.6	0.7	5	4	5
	4	*Ensuring the preceding antenatal consultations are shared among the treating team in a coordinated manner*	4.9	0.4	5	5	5
	5	*Acknowledging the contribution of all professionals from Consultant to bedside nurse to junior doctor*	4.6	1.1	5	4	5
	6	*Information sharing among professionals*	4.8	0.5	5	5	5
	7	*Using 'family held records' to allow families to document meetings , their content, and reflections of understanding*	4.1	0.7	4	4	5
When communicating between professionals…	*8*	*Processes should be put in place by the hospital Trust to strengthen communication between community care providers, hospices, and regional hospitals*	4.6	0.6	5	4	5
	9	*Professionals in the 'team around the child' should be acknowledged and identified early in the child's care pathway*	4.7	0.5	5	4	5
	10	*A case worker/ liaison nurse should be assigned to each complex case to improve coordination across different teams*	4.7	0.6	5	4	5
	11	*A single clinical lead for the child should be defined across all the involved speciality teams*	4.7	0.5	5	4	5
In the process of shared decision	*12*	*We need to be honest with families about medical uncertainty*	4.9	0.3	5	5	5

Confidential: For Review Only

Theme	No.	Statement	Mean score	Standard deviation	Median	IQR 25	IQR 75
making in the child's best interests…	*13*	*There should be a holistic assessment of the child's and families wishes, values and goals to inform more complex decisions later*	4.4	0.7	5	4	5
	14	*We need to, where possible and appropriate, need to listen to what children have to say*	3.7	1.0	4	3	4
	15	*Be aware that children may have motivations separate to what they may say (eg wanting to please their parents)*	4.6	1.0	5	4	5
	16	*Children have rights and these may require independent advocacy even if this means health care professionals disagreeing with their parents*	4.8	0.4	5	5	5
	17	*A holistic assessment of the child's and families wishes, values and goals should include an assessment of their spiritual and religious beliefs*	4.8	0.4	5	5	5
	18	*We should engage families in 'parallel planning' early and routinely in a child's disease course*	4.8	0.5	5	5	5
	19	*Professionals need training to understand the legal framework in which they operate*	4.8	0.4	5	5	5
	20	*Professionals need to be trained in communication techniques*	4.7	0.5	5	4	5
	21	*A holistic assessment of the child's and families wishes, values and goals should be done in partnership with members of the child's MDT [Multi Disciplinary Team]*	4.5	0.7	5	4	5
	22	*We should try to provide options rather than making closed recommendations*	4.5	0.8	5	4	5
	23	*Professionals find it distressing when they are unable to fix medical problems and need supporting when this happens*	4.3	0.8	4	4	5

Confidential: For Review Only

Theme	No.	Statement	Mean score	Standard deviation	Median	IQR 25	IQR 75
	24	*We need to transparently share our own values and goals with families and children*	4.5	0.7	5	4	5
The MDT [Multi Disciplinary Team] should…	*25*	*Be well supported through administrative assistance with preparation and note taking*	4.4	0.6	4	4	5
	26	*Have outcomes recorded in a consistent and transparent fashion*	4.3	0.8	4	4	5
	27	*Be recognised in job plans, given that repeated attendance at MDT [Multi Disciplinary Team] meetings by recognised key professionals is onerous and takes time*	4.6	0.6	5	4	5
	28	*Be held in an appropriate physical environment to enable clarity of discussion*	4.8	0.4	5	5	5
	29	*Be attended by the wider team including hospital, community and hospice representatives*	3.6	1.0	4	3	4
	30	*Ensure that appropriate weight given to all expressed views*	3.0	0.8	3	3	3
	31	*Be chaired by professionals who are trained in chairing such meetings*	4.7	0.5	5	4	5
	32	*Only take place when attempts have been made to understand the child's values and goals*	4.4	0.7	4	4	5
	33	*Be chaired by a professional outside the child's primary clinical team*	4.1	0.7	4	4	5
When managing professional – parental disagreement or conflict…	*34*	*Families should be given realistic honestly held opinions*	4.3	0.7	4	4	5
	35	*We need to better recognise and support mental health issues in families*	4.3	0.7	4	4	5
	36	*We need to prevent the 'threat response' whereby professionals adopt behaviours to avoid contact with families/ each other*	4.8	0.4	5	5	5

Theme	No.	Statement	Mean score	Standard deviation	Median	IQR 25	IQR 75
	37	*The organisation should recognise that (in a rights and consumer based society) any framework that is put in place to improve decision making may not negate conflict and/ or complaint*	4.7	0.8	5	4	5
	38	*Professional-parental disagreement takes resources and time from other patients and so should be an issue of the highest priority for the organisation*	4.1	0.8	4	4	5
	39	*The organisation has a responsibility to convey the challenges of decision making, in the context of patient complexity, to the wider society (local community, NHS leaders, national bodies)*	3.5	1.1	4	3	4
	40	*Families and professionals should receive advice and support on the benefits and risks of social media use*	4.0	1.1	4	4	4
	41	*Standardised information should be provided to families explaining how the decision making process works*	3.6	0.8	4	3	4
	42	*In rare circumstances parental-behavioural contracts are an important tool in addressing disruptive parental behaviour*	3.7	0.8	4	3	4
	43	*A standardised pathway for decision making should set out processes for where disagreement arises*	4.0	1.1	4	4	4
	44	*The CEAG [Clinical Ethics Advisory Group] is an important resource and should be included in any standardised pathway*	3.8	1.3	4	3	4
	45	*There is a need nationally to standardise processes by which 2nd opinions are sought (when?, who? how?, with parental engagement?)*	4.1	1.1	4	4	5

Confidential: For Review Only

Theme	No.	Statement	Mean score	Standard deviation	Median	IQR 25	IQR 75
	46	*External second opinions should be sought from a national peer reviewed speciality MDT [Multi Disciplinary Team] where these are available*	4.3	0.6	4	4	5
	47	*The ward round hand over is an important opportunity to identify evolving issues*	4.7	0.5	5	4	5
	48	*National peer reviewed MDTs [Multi Disciplinary Team] should be convened in specialities where they do not exist*	4.3	1.0	4	4	5
	49	*Gaining an external second opinion is important and should be included in any standardised pathway*	4.4	0.6	4	4	5
	50	*Gaining a local second opinion is important and should be included in any standardised pathway*	4.3	0.8	4	4	5
	51	*A 'traffic light system' whereby a family's behaviour is graded allows earlier identification of conflict and should be included in a standardised pathway to reduce conflict*	4.4	1.0	4	4	5
Clinical psychologists should…	*52*	*Be integrated within all clinical teams*	4.3	1.0	4	4	5
	53	*Support the decision-making process*	4.5	1.0	5	4	5
	54	*Support training in communication techniques*	4.2	1.0	4	4	5
	55	*Routinely explore family goals and values and explicitly share such information with the MDT [Multi Disciplinary Team]*	4.4	0.9	4	4	5
	56	*Have their role better explained to families*	4.4	0.9	4	4	5
	57	*Support training in chairing complex MDT [Multi Disciplinary Team] meetings*	4.2	1.0	4	4	5
	58	*Be involved from the outset in all complex decision-making discussions*	4.4	1.0	5	4	5
In supporting staff…	*59*	*Teams need to adopt process where they can come together to discuss challenging cases*	4.7	0.5	5	4	5

Confidential: For

Theme	No.	Statement	Mean score	Standard deviation	Median	IQR 25	IQR 75
	60	*Teams need to adopt process where individual colleagues are supported*	4.8	0.4	5	5	5
	61	*The organisation should recognise that parental-professional disagreement (conflict) results in poor morale and staff attrition*	4.8	0.5	5	5	5
	62	*The organisation should recognise the physical, mental, and reputational harm done to professionals in extreme cases of professional-parental disharmony*	4.8	0.4	5	5	5
	63	*The organisation should recognise that nursing colleagues are particularly vulnerable due to the requirement for them to be at the bedside 24 hours a day*	4.9	0.4	5	5	5
	64	*The organisation should recognise that HCPs intrinsic desire to 'do the right thing' through leading in complex cases often overrides regard for their personal wellbeing, and increases their vulnerability to experiencing moral distress, compassion fatigue and burnout*	4.2	1.0	4	4	5
	65	*Professionals should take decisions as a team seeking quoracy (consensus within the core team) wherever possible*	4.6	0.6	5	4	5
	66	*Staff have a responsibility themselves to access available support options*	4.2	0.7	4	4	5
	67	*Cross speciality case forums (such as Schwartz rounds) are helpful for different teams to come together to discuss challenging scenarios. While not part of a decision making pathway they should be available for staff aftercare.*	3.8	0.8	4	3	4
	68	*The organisation should provide better opportunities for 1:1 and group peer support*	4.1	0.8	4	4	5

Confidential: For Review Only

Theme	No.	Statement	Mean score	Standard deviation	Median	IQR 25	IQR 75
	69	*The organisation should do more to signpost colleagues to available resources*	4.8	0.4	5	5	5

Table 4: Results after final round of Delphi

Confidential: For Review Only

Discussion

The most notable finding in our study was the uniform degree of consensus across a group of hospital professionals on how best to approach decision making in children with complex care needs. The 5 major themes (standardising the approach to communication with families, communication between professionals, shared decision making, the role of the multidisciplinary team, managing conflict, the role of clinical psychology, and staff wellbeing) resonated with colleagues and provided a useful framework for exploring specific principles. While the level of consensus created by the discussions in the workshop and subsequently measured in all themes was striking, there is perhaps some merit for observing those that carried weight and discussing their implications.

The survey highlights the essential importance of good communication both with the family and between professionals. Concepts such as the 'team around the child', a liaison or 'key worker', and a single defined clinical lead, are not new but do require some committed resource and intent. All too often parental-professional disharmony arises due to misunderstandings or perceived poor co-ordination of care. While the survey talks of a 'standardised' approach to communication, this is not intended to imply a 'one size fits all' solution. Each family is unique and good communication is successful because the needs of each participant are individually recognised.[16] There is a delicate balance between structure and intimate spontaneity that must be achieved in practice.

There was strong agreement that clinicians and parents should work in partnership for the best interests of the child. This is particularly pertinent in the wake of recent court cases [17] where some voices within academic, ethical and legal circles have argued that a shift in authority toward parents in shared decision-making is needed.[18] Participants supported the principle that children have rights that might require independent advocacy. However, while there was strong consensus that transparency was important, professionals were more ambivalent about the importance of this reflecting their own values and goals (statement 24). Finally, there was consensus that good care for children might imply a willingness to consider the child in isolation as well as within a family unit, and that one should not automatically assume that children will share their parents' values.

Although children were therefore recognisedto be central to professionals' considerations, there was also a strong consensus on the importance of discussing the family's wishes (Statements 13, 17, 21 and 22) within a process of shared decision making (Statement 18). While there was an acknowledgement of the importance of seeking parents' views, our survey was not able to adequately explore professionals' insight into the 'lived experience' of a family navigating the distress of caring for a critically ill child in hospital. For example, grading of parental behaviour using a 'traffic light system' (Statement 51) should not be misinterpreted as a judgmental critique of a family's behaviour but rather as an opportunity for professionals to think about their own assumptions and approaches and a hospital to think about whether extra resources are required to assist mediation. Furthermore, while the effects of conflict upon professionals are well reported (statements 61 and 62), we acknowledge that there is less attention to the effect of conflict upon the wellbeing of parents and families.

While there was clear consensus for most statements (mean scores >/ equal 4) there were some where support was more ambivalent. An example of this was Statement 14 that reflected the importance of listening to the child's voice. We interpret this apparent ambivalence as colleagues

https://mc.manuscriptcentral.com/bmjpo

taking a pragmatic view in that children with complex needs often have cognitive and communication deficits, or are ventilated on intensive care, which makes knowing the views of the child challenging. That said, an alternative interpretation might be that some professionals are not appropriately inclusive. We would support future studies that look to how the perspectives of parents and children are represented. Engagement of parents and, especially, children may require research processes, such as deliberative methods, that are less demanding and more resource intense than a Delphi process.[19,20]

There was strong agreement about the importance and structure of the MDT (Multi Disciplinary Team) although there was a predominant view that such a meeting need not be chaired by a professional outside the child's primary clinical team (statement 33). Interestingly there was some ambivalence expressed regarding the inclusivity of these meetings with regards to the attendance of the wider team and the giving of weight to all views (statements 29 or 30). This might reflect a pragmatic view around the logistics of holding such meetings in a timely fashion, or an adherence to intra-professional considerations of hierarchy and expertise, and where ultimately professionals regard decision making to lie. In our view MDT meetings with or without families form the vital platform upon which clinical decisions are taken. They should involve all key professionals. Yet it should be acknowledged that they therefore require much planning, should be recognised in job plans and be granted sufficient resources.

When things go wrong, complaint and conflict too often arise. These are challenging for both families and members of the clinical team. There was a tacit acknowledgment that, despite best intentions and optimal circumstances, conflict will occasionally still arise. Organisations should give conflict resolution the highest priority and consider standardised approaches to clinical ethics consultation and external second opinions. Referral for external second opinions has been a consistent recommendation for reducing conflict in many previous publications.[12,21,22] However, in our study, while there was a strong consensus for such processes to be standardised at a national level (statement 45), there was slightly more ambivalence around these being introduced as a component of an automatic conflict resolution pathway at a local level (statement 49 & 50). Participants in our study were also supportive of involving national peer review MDTs where these were available (statement 46) or establishing such bodies where they were not (statement 48).

There was consensus for Clinical Psychology support to be integrated within clinical teams, and for them to take an active role in exploring family goals and values and supporting complex decision-making discussions. Finally, there was strong agreement around the importance of supporting staff, and that, in situations of professional-parental disharmony, poor morale and staff attrition result. The deleterious impact on clinicians of difficult relationships and challenging dilemmas in practice has been previously noted.[23,24,25] A primary focus on individual resilience may miss the importance of investment in institution wide structures.[26] Health care professionals' intrinsic desire to 'do the right thing' through leading in complex cases may over-ride their regard for their personal wellbeing, and increase their vulnerability to experiencing moral distress, compassion fatigue and burnout.

Limitations

Importantly, we did not consider the views of either parents or children in this Delphi survey. This is because the survey was conceived as a quality improvement exercise that sought to share best practice between professionals in response to specific institutional challenges. While the exercise would have been improved with the inclusion of parental and children's views, a comprehensive approach would involve a widespread consultation, probably using a more interactive and engaging technique than a Delphi ranking. Unfortunately such an expanded project was beyond our scope and

Confidential: For Review Only

resources. Further consensus work will need to ensure that the statements included here provide a solid basis for a model for partnership between hospital staff, parents and children, as well as the range of agencies that are also involved in complex care within the community: for example hospice providers, social work and third sector agencies.

Given the high degree of consensus shown it is worth considering if this consensus was genuine. Our statistical analysis showed that dropping out of the Delphi was not due to being a statistical outlier, suggesting the process did not discourage dissenters from taking part. Nevertheless, 74.5% of those approached did not engage with the process at all. Although the level of engagement seems broadly comparable with similar Delphi studies[27,28] (and accounting for the fact that non-engagement is a problem common to many types of research), we cannot discount the idea that a proportion of these staff did not value the aims of the study.

Conclusion

The number of children with complex care needs in our hospitals is increasing. Our study of a large mixed group of healthcare professionals in a tertiary children's hospital demonstrates strong consensus across 5 key themes towards developing best practice in decision making in children with complex care needs: standardising the approach to communication with families, communication between professionals, shared decision making, the role of the multidisciplinary team, managing conflict, the role of clinical psychology, and staff wellbeing. Within each of these themes there are notable elements of practice that health care teams might engage with to better support the patients and families in their care. In some situations, a comprehensive adoption of a standardised approach, might mitigate against professional-parental disharmony.

References:

[1] Brenner M, Kidston C, Hilliard C, *et al.* Children's complex care needs: a systematic concept analysis of multidisciplinary language. *Eur J Pediatr*. 2018;177(11):1641-1652.
[2] Fraser LK, Gibson-Smith D, Jarvis, S *et al*. Estimating the current and future prevalence of life-limiting conditions in children in England. *Palliat Med*. 2021;35(9):1641-1651.
[3] Forbat L, Sayer C, McNamee P, *et al.* Conflict in a paediatric hospital: a prospective mixed-method study. *Arch Dis Child* 2016; 101(1):23-7
[4] Azoulay E, Timsit JF, Sprung CL. Prevalence and factors of intensive care unit conflicts: the conflicus study. *Am J Respir Crit Care Med* 2009;180:853-60
[5] Department of Health (2003) Building on the Best: Choice, Responsiveness and Equity in the NHS, Cmnd. 6079 (London, The Stationery Office): 63
[6] Children Act 1989 s.1(1)
[7] Streuli JC, Anderson J, Alef-Defoe S, *et al.* Combining the best interest standard with shared decision-making in paediatrics—introducing the shared optimum approach based on a qualitative study. *Eur J Pediatr* 2021;180**:** 759–766
[8] Birchley G, Deciding together? Best interests and shared decision-making in paediatric intensive care. *Health Care Anal* 2014; 22:203–222
[9] The Gard Foundation, What is Charlie's Law? Available at: https://thecharliegardfoundation.org/ Accessed 25th April 2022
[10] Kanthimathinathan HK, Plunkett A, Scholefield BR *et al.* Trends in long-stay admissions to a UK paediatric intensive care unit *Arch Dis Child* 2020;105:558-562
[11] Gerada C (editor), *Beneath the White Coat: Doctors, Their Minds and Mental Health* (Routledge 2020)

Confidential: For Review Only

[12] Linney M, Hain RDW, Wilkinson D, *et al* Achieving consensus advice for paediatricians and other health professionals: on prevention, recognition and management of conflict in paediatric practice *Arch Dis Child* 2019; 104:413-416.
[13] Forbat L, Teuten B, Barclay S, Conflict escalation in paediatric services: findings from a qualitative study *Arch Dis Child* 2015;100:769-773.
[14] Murphy MK , Black NA , Lamping DL, *et al.* Consensus development methods, and their use in clinical Guideline development. *Health Technol Assess* 1998; 2:i–iv.1-88.
[15] Sterne JAC, Cox DR, Smith GD. *Sifting the evidence—what's wrong with significance tests? Another comment on the role of statistical methods* BMJ 2001; 322 :226.
[16] Ward P. Trust and communication in a doctor-patient relationship: a literature review. *Arch Med.* 2018;3(3):36.
[17] *Yates and Gard v Great Ormond Street Hospital* [2017] EWCA Civ 410
[18] Auckland, C and Goold, I (2020) Re-evaluating 'best interests' in the wake of Raqeeb v Barts NHS Foundation Trust & Anors *Mod Law Rev* 83(6) 1328-1342.
[19] Stark, L., Robinson, M.V., Gillespie, A. et al. Supporting mental health and psychosocial wellbeing through social and emotional learning: A participatory study of conflict-affected youth resettled to the U.S. *BMC Public Health* 2021; 21, 1620.
[20] Kim SY1, Wall IF, Stanczyk A, De Vries R, Assessing the public's views in research ethics controversies: deliberative democracy and bioethics as natural allies. *J Empir Res Hum Res Ethics*. 2009; 4(4):3-16.
[21] Austin L, Huxtable R. Resolving Disagreements about the Care of Critically Ill Children: Evaluating Existing Processes and Setting the Research Agenda In: Goold I, Herring J, Auckland C (eds) Parental Rights, Best Interests and Significant Harms: Medical Decision-Making on Behalf of Children Post-Great Ormond Street Hospital v Yates (Oxford: Hart Publishing, 2019)
[22] Nuffield Council on Bioethics, Disagreements in the care of critically ill children (Bioethics Briefing Note) (London: Nuffield Council on Bioethics, 2019)
[23] Gerada C (editor), Beneath the White Coat: Doctors, Their Minds and Mental Health (Routledge 2020); Barclay S. Recognizing and managing conflict between patients, parents and health professionals. *Paediatr Child Health* 2016; 26(7):314-315
[24] Carmela Mento, Maria Catena Silvestri, Antonio Bruno, *et al.* Workplace violence against healthcare professionals: A systematic review, *Aggress Violent Behav* 2020; 51:101381
[25] Knickle K, McNaughton N, Downar J. Beyond winning: mediation, conflict resolution, and non-rational sources of conflict in the ICU. *Crit Care* 2012; 16:308
[26] Johal HK, Prout R, Birchley G *et al.* We should move away from a focus on individual resilience towards building resilient systems *BMJ* 2021; 375:2737
[27] Murray MJ, Ruffle A, Lowis S, *et al* Delphi method to identify expert opinion to support children's cancer referral guidelines *Arch Dis Child* 2020; 105:241-246
[28] Ekberg S, Herbert A, Johns K, *et al.* Finding a way with words: Delphi study to develop a discussion prompt list for paediatric palliative care. *Palliat Med* 2020; 34(3):291-299

Confidential: For Review Only
